# Health Tract, No. 209

**Published:** 1865

**Authors:** 


HEALTH TRACT, No. 209.

### Summer Recreations.

FOUR young men, knapsacked in the old Continental style, passed along the other day, bound for the White Mountains; they were a hale, jolly-looking set, intending to travel about twenty miles a day, resting from nine A.M. to five P.M.; they lived chiefly on bread and milk, bountifully procured on the road at small cost. This is the true philosophy of summer recreation; and let clerks, clergymen, lawyers, judges, students, and other sedentary persons make a note of it, for it is an example well worth following. Such a "trip," from the middle of August to the first of October, over the hills of New-England, the Catskill Mountains, or the Adirondacks, would be glorious. We had a taste of this ourselves, just twenty years ago this autumn, beginning at Menai Bridge, through Bangor Walls, the famous slate quarries, over to Dublin and Donnybrook Fair, up through Ireland to Belfast, with its linen factories; the Giant's Causeway; over to the Banks of Bonny Doon, Glasgow, Edinburgh, Abbotsford, and the famous castle or church there, which we first saw by the aid of a tin lantern, too impatient to wait until next morning, being paid for our hurry by seeing a drove of sleepy sheep in the inclosure; thence to Berwick-upon-Tweed, which divides Scotland and England; thence by steamer to great London, where we footed it indefinitely, *ad infinitum*, all over. These are perigrinations of some account; and the memory of them is inexpressibly dear. How luscious the draughts of what we saw, and felt, and heard, and such an appetite—such glorious sleep! We were chuck full of life, and frolic, and fun, wanted to run foot-races on the road every day, with our younger and more sedate brother, "Dr. Sam." Poor fellow, a dozen years dead. Dignified people always do die before their time. This way of being as sober as a judge isn't wholesome; it clogs up the wheels of life, and stops them prematurely. We poked at him some of our mother's philosophy, who never did any thing in her life until she had satisfactorily answered the question, "What's the use?" Why, it's fun to run a race, especially if you are blindfolded, and making for a tree on the sea-shore, to get there first. Suppose it is "infra dig," nobody knows us; and if they did, it is none of their business whether we walk, run, or roll over, and with that we turned a double summerset, while he called for a smelling-bottle. Reader, we believe in fun more than physic, hence we "still live," and lively as a cricket too!

